# Adaptation and evaluation of the measurement properties of the Brazilian version of the Self-efficacy for Appropriate Medication Adherence Scale^1^

**DOI:** 10.1590/1518-8345.0167.2692

**Published:** 2016-05-17

**Authors:** Rafaela Batista dos Santos Pedrosa, Roberta Cunha Matheus Rodrigues

**Affiliations:** 2 Doctoral Student, Faculdade de Enfermagem, Universidade Estadual de Campinas, Campinas, SP, Brazil; 3 PhD, Full Professor, Faculdade de Enfermagem, Universidade Estadual de Campinas, Campinas, SP, Brasil

**Keywords:** Validation Studies, Medication Adherence, Self-efficacy

## Abstract

**Objectives:**

to undertake the cultural adaptation of, and to evaluate the measurement properties of, the Brazilian version of the Self-efficacy for Appropriate Medication Adherence Scale in coronary heart disease (CHD) patients, with outpatient monitoring at a teaching hospital.

**Method:**

the process of cultural adaptation was undertaken in accordance with the international literature. The data were obtained from 147 CHD patients, through the application of the sociodemographic/clinical characterization instrument, and of the Brazilian versions of the Morisky Self-Reported Measure of Medication Adherence Scale, the General Perceived Self-Efficacy Scale, and the Self-efficacy for Appropriate Medication Adherence Scale.

**Results:**

the Brazilian version of the Self-efficacy for Appropriate Medication Adherence Scale presented evidence of semantic-idiomatic, conceptual and cultural equivalencies, with high acceptability and practicality. The floor effect was evidenced for the total score and for the domains of the scale studied. The findings evidenced the measure's reliability. The domains of the Brazilian version of the Self-efficacy for Appropriate Medication Adherence Scale presented significant inverse correlations of moderate to strong magnitude between the scores of the Morisky scale, indicating convergent validity, although correlations with the measure of general self-efficacy were not evidenced. The validity of known groups was supported, as the scale discriminated between "adherents" and "non-adherents" to the medications, as well as to "sufficient dose" and "insufficient dose".

**Conclusion:**

the Brazilian version of the Self-efficacy for Appropriate Medication Adherence Scale presented evidence of reliability and validity in coronary heart disease outpatients.

## Introduction

Although it is highly prevalent worldwide^(^ 1 ^)^, recent studies indicate that the advances in the treatment of Coronary Heart Disease (CHD) have contributed to a decline observed in the rates of hospitalization and in mortality through Acute Myocardial Infarction (AMI)^(^ 1 ^-^ 2 ^)^. Evidence demonstrates the efficacy of the use of cardioprotective therapy (Beta blockers, Angiotensin-Converting Enzyme Inhibitors (ACE-inhibitors) or Angiotensin-Receptor Blockers (ARBs), statins and antiplatelets) in the secondary prevention of CHD, the combined use of this therapy being widely recommended^(^ 3 ^)^. In addition to this, the use of these medications was associated with the reduction in the relative risk of death through CHD ^(^ 2 ^-^ 3 ^)^. In conjunction with the cardioprotective drugs, the use of medications for relieving symptoms is also related to the patients' greater tolerance to the symptoms of CHD^(^ 1 ^,^ 3 ^)^. As a result, the prognosis of CHD is closely related to adherence to the cardioprotective medications and to medications which relieve the symptoms. 

Adherence, is defined as the extent to which the patients follow the guidance for the treatment which they are provided with by the doctor and/or other health professionals^(^ 4 ^)^. Therefore, nonadherence occurs when the patient's behavior does not coincide with these recommendations^(^ 5 ^)^.

For better comprehension of the construct of medication adherence, some theories have been utilized^(^ 6 ^)^, among them Bandura's Social-Cognitive Theory; self-efficacy is this theory's central concept. Self-efficacy may be defined as a belief or trust that one can successfully undertake a specific action, in order to achieve the desired result^(^ 7 ^)^.

The complexity of medication adherence goes beyond understanding the construct itself, and encompasses the extreme difficulty involved in its accurate measurement. Various methods are available in the literature^(^ 8 ^)^, including the self-reported scales. Among the reliable and valid tools for evaluation of self-efficacy, for the behavior of adherence, the Self-efficacy for Appropriate Medication Adherence Scale (SEAMS)^(^ 9 ^)^, an American scale, stands out. This was constructed in order to assess self-efficacy for medication adherence among individuals with low educational levels. This scale presented adequate measurement properties, when applied in 436 patients with CHD and other comorbidities. The authors do not know of any self-reported instruments for the measurement of self-efficacy, for the behavior of medication adherence, in the Brazilian context. 

As a result, this study's objectives were to undertake the cultural adaptation of the SEAMS to Brazilian Portuguese and assess its measurement properties among patients with CHD being treated on an outpatient basis. The specific objectives were to ascertain practicality, acceptability, ceiling and floor effect, reliability and convergent validity, and known groups validity. This research's findings may guide more efficacious conducts in regard to strengthening the self-efficacy for adherence to drug therapy among coronary heart disease (CHD) patients.

## Methods

### The methodological procedure of cultural adaptation 

The following stages were used for the process of translation and adaptation: translation - following the obtaining of consent from the author, the SEAMS was translated to Portuguese by two independent bilingual translators whose mother tongue is Brazilian Portuguese, only one of these being informed about the scale's concepts and objectives^(^ 10 ^)^; synthesis of the translations - the translated versions (T_1_ and T_2_) were analyzed and compared by the researchers and by a professional mediator-translator^(^ 10 ^)^. The discrepancies were analyzed until consensus was obtained - the translated version of the SEAMS (T_1-2_); back translation - the translated version of the SEAMS was translated back into English by two other independent bilingual translators, who had not participated in the first stage, whose mother language was English and who were not aware of the instrument's concepts/purposes. At the end of this stage, the following versions were obtained - back-translation 1 (BT_1_) and back-translation 2 (BT_2_); evaluation by a Committee of Judges: made up of five bilingual experts who evaluated the translated version in relation to the *semantic and idiomatic, cultural and conceptual equivalencies* ^(^ 10 ^)^ and pre-test - the adapted version was applied in 10 patients with CHD being treated on an outpatient basis. After responding to each item of the scale, the participants were interviewed in order to investigate the difficulties perceived in relation to the understanding of the statements and the response scale, as well as to detect terms which were difficult to understand. 

### Methodological procedures for evaluation of the measurement properties 

### The research locale

The study was undertaken in the cardiology outpatient center - Ischemic Heart Disease subspeciality - of a teaching hospital in the nonmetropolitan region of the State of São Paulo.

## Subjects

A total of 147 patients took part in this study, with previous clinical manifestation of unstable angina and/or acute myocardial infarction, receiving treatment on an outpatient basis, with a period of over six months since the last event, with a view to excluding those patients known to be clinically unstable, whose drug therapy is frequently modified, which could influence their behavior of adherence to the drug treatment^(^ 2 ^-^ 3 ^)^. Patients in continuous use of cardioprotective drugs and/or drugs for relieving the symptoms for at least two months were included, as this is a period in which the patient is familiarized with the drug treatment prescribed. Those patients who presented inability for effective verbal communication were excluded. 

### Sampling procedure and sample size 

The sample was made up of patients attended in the above-mentioned service, enrolled non-randomly, in October 2013 - January 2014. The sample size was calculated using the Spearman correlation coefficients, between the SEAMS scores and the measures of medication adherence, obtained in a pilot-study (n=15). Considering correlation coefficients between 0.30 and 0.40, and values of α=0.05 and beta=0.9, the minimum number of 105 subjects was calculated. Losses being foreseen, the sample size was extended to 147 subjects. 

### Data collection procedure 

The data were obtained by the researcher, individually, in a private environment, in accordance with the stages shown below. 

- First stage: consent to participate in the study was obtained through the signing of the Terms of Free and Informed Consent (TFIC), and information was collected regarding sociodemographic and clinical characterization, through interview and consulting medical records. The following were applied: the adapted version of SEAMS, the Brazilian versions of the Morisky Self-Reported Measure of Medication Adherence Scale (MMAS-4) and of the General Perceived Self-efficacy Scale (GSE), as well as measurements of adherence - proportion of adherence and global evaluation of medication adherence.

- Second stage: the Brazilian version of the SEAMS was reapplied (retest) in a proportion of the subjects who participated in the application (test), in similar conditions, with an interval of fifteen days between the first and second application. In this stage, only those participants whose return was arranged in the above-mentioned service took part (n=34).

### Data collection instruments 

Instrument for sociodemographic and clinical characterization: the instrument constructed and subjected to content validity in a previous study was used^(^ 11 ^)^.

Definition of the drug therapy evaluated: the drug therapy evaluated was related to reduction in CHD's morbidity and mortality - lifesaving therapy - (that is, ACE-inhibitors, ARBs, Beta blockers, antiplatelet drugs and statins) and two other drugs which improve the signs and symptoms associated with coronary heart disease (that is to say, digitalis, diuretics and nitrates).

Morisky Self-Reported Measure of Medication Adherence Scale (MMAS-4): an instrument constituted by four questions relating to adherence to the drug treatment, assessing forgetting, carelessness, interruption of the use of the drug as a result of perceiving improvement, and interruption of the therapy due to perceiving worsening in the clinical situation^(^ 12 ^)^. The Brazilian version of the Morisky scale will be used^(^ 13 ^)^. In the Brazilian version, a Likert-type response scale was used, of 4 to 5 points, varying from (1) Never to (5) Daily; (1) Never to (5) Always and (1) Never to (4) Always. The sum of the responses to the four items generates a score between 4 and 18; higher scores indicate low adherence; lower scores, high adherence. 

- Self-reported measure of adherence: according to proportion of medication adherence and global evaluation of medication adherence. 

Proportion of medication adherence: this instrument is made up of four fields covering: 1. Description of name, dose and how to take all the prescribed drugs; 2. Description of the drugs used on the day before the interview, by dose and how they are to be taken; 3. Drugs used the previous week and 4. Drugs used in the month prior to the interview. Fields 2 and 3, referent to the previous day and week, respectively, aimed to obtain more accurate responses through minimization of the memory bias. Only data from field 4, referent to the use of medication in the previous month will be used for calculating the proportion of adherence. The adherence was calculated based on the doses omitted, according to the following calculation: [(doses prescribed - doses missed) x 100/doses prescribed]^(^ 14 ^)^. The variable of adherence was treated as continuous (percentage of the doses taken in the month immediately preceding the interview) and categorical: *appropriate dose* (dose used ≥80% of the dosage prescribed) and *insufficient dose* (dose used <80% of the dosage prescribed). For the patients who made use of more than one medication, the final proportion of adherence was calculated by the mean of the percentages of adherence to each medication. The participants who made use of a dose which was above that prescribed had their values converted to the corresponding rates below 100%; that is, the participant with 120% adherence, as she exceeded complete adherence by 20%, would correspond to a value of 80% of adherence. 

- Global evaluation of adherence: in this measurement, besides the proportion of taking of medications, the way in which these are taken, the frequency and the necessary care for administering the medications was evaluated, taking into account the association with time markers: fasting, breakfast, lunch, dinner, and at bedtime. Therefore, the adherence, according to the dosage of the medications and care taken, termed global evaluation of adherence, was evaluated based in the following classification: Group I - appropriate dose and care for the prescription; Group II - correct dose and inadequate care; Group III - incorrect dose and inadequate care, and Group IV - inadequate dose and inadequate care. "Inadequate care" is considered to be the use of one or more medications, in which how they should be taken (number and frequency of medications) and association with time markers (fasting, breakfast and lunch), are not in accordance with the medical prescription. The participants classified in Group I were considered "adherent" and those classified in the other groups, as "nonadherent"^(^ 15 ^)^.

- General Perceived Self-efficacy Scale **(**GSE): an instrument created by Schwarzer and Jerusalem^(^ 16 ^)^, which is unidimensional and made up of 10 items, which refer to how to deal with success in a specified situation. The participant responds to the instrument through a five point Likert response scale which varies from 1 (totally disagree) to 5 (totally agree). The total score has a variation from 10 to 50. A high score signifies a high perception of self-efficacy. The version adapted to Brazilian Portuguese was used^(^ 17 ^)^.

- Self-efficacy for Appropriate Medication Adherence Scale (SEAMS): this is made up of 13 items, divided in two domains: *self-efficacy for taking medications in difficult circumstances* (07 items) and *self-efficacy to continue to take the medication, under uncertain circumstances* (06 items). In order to answer the instrument, the participant must indicate his or her level of confidence in relation to the correct use of the medications; the response can vary from 1 to 3, with 1 (not confident), 2 (little confident), and 3 (very confident). The total score, which consists of the sum of the responses, can vary between 13 and 39; the higher the score, the greater the self-efficacy for adherence to the drug treatment^(^ 9 ^)^.

### Analysis of the data 

- Analysis of the Content Validity: the Content Validity Index (CVI) was used for evaluation of the semantic-idiomatic, conceptual and cultural equivalencies. This measures the proportion of judges who are in agreement regarding the items and general aspects evaluated^(^ 10 ^)^. The items' relevance and representativity was evaluated, through a Likert-type scale with scores varying between 1 and 4 (1= not relevant or not representative, 2= requiring major revision in order to be representative, 3= requiring minor revision in order to be representative, 4= relevant or representative). The CVI was calculated through the sum of agreement of the items which received scores of "3" or "4", divided by the total number of responses. The items with scores of "1" or "2" were revised. 

- Descriptive analysis, of the reliability and validity of the Brazilian version of the SEAMS: the collected data were inserted into an electronic spreadsheet in the Statistical Package for the Social Sciences (SPSS) program, version 17.0, for Windows, for the statistical analyses. 

- Descriptive analysis: tables of frequency and measurements of position and dispersion for the clinical and sociodemographic characterization data and for the scores of the scales used were made. Practicality was evaluated through the mean time spent in the application and the acceptability by the percentage of participants who responded to all the items^(^ 18 ^)^. The floor effect, which is equivalent to the 10% of the scale's worst possible results, and the ceiling effect, which corresponds to the 10% of the scale's best possible results, were evaluated^(^ 19 ^)^.

- Evaluation of reliability: the Cronbach alpha coefficient was used to calculate the internal consistency, with a Cronbach alpha of >0.70 being established as evidence of satisfactory internal consistency^(^ 20 ^)^. In order to evaluate the stability of the measure, the Intraclass Correlation Coefficient (ICC) was used, with ICC >0.7 being considered satisfactory^(^ 21 ^)^.

- Calculation of the construct validity: the convergent construct validity and the validity of known or contrasted groups were tested. In order to estimate the convergent construct validity, Spearman's correlation coefficient was used in order to test the correlation between the scores of the Brazilian versions of the SEAMS, the GSE and the MMAS-4, considering the coefficients of <0.30 to be of weak magnitude, those between 0.30 and 0.50 to be of moderate magnitude, and those >0.50 to be of strong magnitude^(^ 22 ^)^. Negative correlations of strong magnitude were hypothesized between the domains of the Brazilian version of the SEAMS and the total score for the MMAS-4, and significant positive correlations of strong to moderate magnitude between the SEAMS and the GSE. 

The construct validity of known or contrasted groups was tested through the use of the Mann-Whitney test, in order to ascertain the instrument's capacity to distinguish between the participants classified as *appropriate dose* or *insufficient dose,* according to the self-reported measurement of proportion of adherence, as well as those considered to be *adherent* or *nonadherent* to the drug therapy, according to the global evaluation of adherence. It was hypothesized that the participants classified as "nonadherent" and "insufficient dose" would present the lower self-efficacy for medication adherence, according to the proportion of medication adherence. 

A level of significance of 5% was adopted. 

### Ethical aspects 

The study was approved by the university's Research Ethics Committee (Opinion N. 254.844/2013) and all the patients enrolled signed the TFIC.

## Results

### Methodological procedure of cultural adaptation 

The results of the content validation (CVI) evidenced between 0.80 and 1.0 in 11 of the 13 items evaluated. Only items 11 and 12 obtained CVI= 0.60, these being revised in order to obtain consensus between the judges. However, some of the experts made suggestions regarding the presentation of the instrument, which were taken into account. As a result, the design was altered and the numbering was removed from the response scale, this being considered not to be important for the respondents. The Brazilian version of the SEAMS was evaluated by the Committee of Judges a second time, and submitted to the pre-test stage. In this stage, the respondents reported understanding the items, and denied difficulties for interpreting the response scale.

### Descriptive evaluation and evaluation of reliability, and construct validity

Sociodemographic and clinical characterization 

A predominance of men was observed (68.0%), with a mean age of 59.9 (Standard-Deviation - sd = 9.6) years old, economically inactive (72.8%), with a mean family income of 2.7 (sd=1.1) Minimum-Salaries (MS)/month (Table 1).

**Table 1:** - Sociodemographic and clinical characteristics of the CHD patients (n=147). Campinas, SP, Brazil, 2014

Variable	%	Mean (sd)*	Median	Variation	
Sex					
	Male	68.0			
Age		59.9 (9.6)	60.0	34-84	
Education - in years (n=152)		5.3 (3.4)	4.0	0-16	
Marital situation					
	Married/cohabiting	69.1			
	Single	12.2			
	Separated/divorced	10.9			
	Widowed	6.8			
Employment status					
	Inactive	72.8			
	Active	23.1			
	Housewife/husband	4.0			
Family income (in MS*)		2.7 (1.1)	3.0	0-5	
Characterization of the coronary heart disease					
	Infarction of the myocardium	83.7			
	Unstable angina	13.6			
Number of previous AMIs† (n=147)		1.2 (0.8)	1.0	0-5.0	
Number of associated symptoms		1.7 (1.5)	1.0	0-5.0	
Signs and symptoms (in the last months)					
	Precordialgia	38.8			
	Dyspnea	32.0			
	Arrhythmia	22.4			
	Syncope	0.7			
Number of associated clinical conditions and/or risk factors		2.9 (1.2)	3.0	0-6.0	
	Systemic Arterial Hypertension (SAH)	94.6			
	Dyslipidemia	65.3			
	Smoking tobacco	67.3			
	Diabetes mellitus (DM)	44.9			
	Obesity (BMI>30kg/m2)	10.2			
Treatment					
	Angioplasty and/or surgical revascularization	55.1			
	Clinical	44.9			
Number of medications in use		6.4 (1.9)	6.0	2-12	

*MS= Minimum-salary, of R$724,00, Brazil, 2014; †AMI - Acute Myocardial Infarction

The majority of the patients (83.9%) had been diagnosed with Myocardial Infarction (MI) (in isolation or associated with post-MI angina) and 2.9 (SD=1.2), with associated clinical conditions and/or risk factors. All the patients reported symptoms in the month prior to the interview, with a mean of 1.7 (sd=1.5) associated symptoms. The mean use of 6.4 (sd=1.9) medications per day was observed. 

### Practicality, acceptability and ceiling and floor effects

The results suggest that the Brazilian version of the SEAMS is an instrument which is easy to apply, with a mean application time of 3 minutes (sd=0.5). All the participants responded to all the items of the SEAMS, which shows the high acceptability of the scale. The analysis of the mean and median values of the total score of the Brazilian version of the SEAMS showed high self-efficacy for medication adherence. The evaluation of the ceiling and floor effects indicated a ceiling effect for the total score and for the domains of the SEAMS (Table 2). 

**Table 2:** - Descriptive analysis of the domains and ceiling and floor effects of the Self-efficacy for Appropriate Medication Adherence Scale (n=147). Campinas, SP, Brazil, 2014

SEAMS* - Domains	N. of items	Mean (sd)	Median	Variation observed	% Floor	% Ceiling
Self-efficacy for taking medications, under difficult circumstances	7	20.2 (1.9)	21.0	9-21	0.0	83.7
Self-efficacy for continuing to take medications when the circumstances which permeate this action are uncertain	6	17.2 (1.9)	18.0	7-18	0.0	83.0
Total score	13	37.3 (3.5)	39.0	17-39	0.0	79.6

*Self-efficacy for Appropriate Medication Adherence Scale (SEAMS).

### Reliability 

The analysis indicated satisfactory internal consistency for the total score and domains of the SEAMS - alpha cronbach of 0.8 for the domain of *Self-efficacy for taking medications, under difficult circumstances* and of 0.9 for the domain of *Self-efficacy for continuing to take medications when the circumstances which permeate this action are uncertain*, and of 0.92 for the total score. Satisfactory Intraclass Correlation Coefficient (ICC) scores were calculated for the domains and total score of the Brazilian version of the SEAMS (Table 3).

**Table 3:** - Analysis of the reliability of the Brazilian version of the Self-efficacy for Appropriate Medication Adherence Scale (n=147). Campinas, SP, Brazil, 2014

SEAMS* - Domains	Cronbach alpha	Item/total correlation	Alpha - If item deleted	ICC^†^	CI95%^‡^
Self-efficacy for taking medications, under difficult circumstances	0.8			0.9	[0.7-0.9]
Item 1		0.5	0.8		
Item 2		0.6	0.8		
Item 3		0.7	0.8		
Item 4		0.6	0.8		
Item 6		0.6	0.8		
Item 7		0.7	0.8		
Item 8		0.6	0.8		
Self-efficacy for continuing to take medications when the circumstances which permeate this act are uncertain	0.9			1.0	[0.9-1.0]
Item 5		0.5	0.9		
Item 9		0.8	0.9		
Item 10		0.8	0.9		
Item 11		0.8	0.9		
Item 12		0.8	0.9		
Item 13		0.7	0.9		
Total score	0.92				

*Self-efficacy for Appropriate Medication Adherence Scale - SEAMS; † Intraclass Correlation Coefficient (ICC); ‡confidence interval of 95%.

### Construct Validity

### Convergent validity 

Significant inverse correlations of moderate to strong magnitude were observed between the total score and the domains of the Brazilian versions of the SEAMS and the MMAS-4. Significant correlations were not observed between the scores of the Brazilian versions of the SEAMS and the GSE. 

**Table 4:** - Spearman correlation coefficients between the scores of the Brazilian versions of the Self-efficacy for Appropriate Medication Adherence Scale, the Morisky Self-Reported Measure of Medication Adherence Scale and the General Perceived Self-efficacy Scale (n=147). Campinas, SP, Brazil, 2014

Brazilian version of the SEAMS*	Measure of medication adherence	Measure of general self-efficacy	
	Brazilian version of the MMAS-4	Brazilian version of the GSE^†^	
	r^‡^	R	
Domain 1 - Self-efficacy to take medications, under difficult circumstances	-0.54†	0.12	
	p<0.0001	p=0.128	
Domain 2 - Self-efficacy to continue to take medications when the circumstances that permeate this action are uncertain	-0.43†	0.22	
	p<0.0001	p=0.0063	
Total score	-0.53^†^	0.22	
	p<0.0001	p=0.0071	
			

*Self-efficacy for Appropriate Medication Adherence Scale (SEAMS); †General Perceived Self-efficacy Scale (GSE); ‡r= correlation coefficient.

### Validity of known or contrasted groups 

The findings evidenced that the Brazilian version of the SEAMS was able to discriminate between patients who adhered, and those who did not, to the medication therapy, according to the global evaluation of the adherence - which considers, besides how the medication is to be taken (dose, form, frequency and how long for), the care for taking the medications. The data showed that, in both the domains and total score of the SEAMS, the score was significantly greater among those who adhered to the medications, in comparison with the nonadherent group, indicating greater self-efficacy for drug adherence in the adherent group, as previously hypothesized. In the same way, the Brazilian version of the SEAMS discriminated self-efficacy among patients categorized as adequate dose and those considered as insufficient dose, according to the proportion of drug adherence, with higher scores in the SEAMS being observed among those patients categorized as adequate dose in domains 1 and 2 (p=0.0051 and p=0.0125, respectively) and total score (p=0.0012) of the SEAMS, when compared with those with insufficient dose (Table 5).

**Table 5:** - Comparison between the scores of the Brazilian version of the Self-efficacy for Appropriate Medication Adherence Scale, according to the global evaluation of medication adherence (n=147). Campinas, SP, Brazil, 2014

Domains of the SEAMS	Global evaluation of adherence	n	Mean	Minimum	Q1	Median	Q3	Maximum	p-value^‡^
Domain 1*	Adherents	87	20.6 (1.4)	9.0	21.0	21.0	21.0	21.0	<0.0001
	Non-adherents	60	19.5 (2.2)	13.0	18.5	21.0	21.0	21.0	
Domain 2†	Adherents	87	17.6 (1.3)	8.0	18.0	18.0	18.0	18.0	0.0026
	Non-adherents	60	16.5 (2.5)	7.0	16.0	18.0	18.0	18.0	
Total score Total	Adherents	87	38.2 (2.6)	17.0	39.0	39.0	39.0	39.0	<.0001
	Non-adherents	60	36.0 (4.3)	22.0	34.0	38.0	39.0	39.0	
	Proportion of adherence								
Domain 1	Adequate dose	133	20.3 (1.7)	9.0	21.0	21.0	21.0	21.0	0.0051
	Insufficient dose	14	18.9 (2.6)	14.0	18.0	20.0	21.0	21.0	
Domain 2	Adequate dose	133	17.4 (1.6)	8.0	18.0	18.0	18.0	18.0	0.0125
	Insufficient dose	14	15.2 (3.8)	7.0	13.0	17.5	18.0	18.0	
Total score	Adequate dose	133	37.7 (3.0)	17.0	38.0	39.0	39.0	39.0	0.0012
	Insufficient dose	14	34.1 (5.9)	22.0	33.0	36.5	39.0	39.0	

*Self-efficacy for taking medications, under difficult circumstances; † self-efficacy for continuing to take medications when the circumstances that permeate this action are uncertain; ‡ Mann-Whitney comparison test.

## Discussion

In this study, the cultural adaptation of the SEAMS was undertaken, and the measurement properties of the Brazilian version of the SEAMS were investigated. The SEAMS is an instrument constructed with the purpose of measuring self-efficacy for medication adherence. The methodological procedure of cultural adaptation was undertaken in CHD patients, with the semantic-idiomatic, conceptual and cultural equivalencies of the Brazilian version of the SEAMS being determined. 

A ceiling effect was observed for the total score and for both domains, indicating that the Brazilian version of the SEAMS may not be sensitive for detecting improvement of self-efficacy. However, the Brazilian version of the SEAMS may be potentially sensitive and responsive to measuring worsening, as the floor effect was not observed. One possible explanation for this finding may be related to the instrument's response scale, whose highly similar options may not have made it possible for participants to differentiate the alternatives. In previous studies^(^ 9 ^,^ 23 ^)^, in which the SEAMS was applied, the evaluation of the instrument's ceiling and floor effect is not found. The present study's findings need to be ratified, as they imply the limitation of its use in experimental studies in order to evaluate the effect of interventions for the strengthening of self-efficacy, for medications adherence. 

The majority of the domains of the SEAMS presented evidence of internal consistency, with the Cronbach alpha oscillating between 0.85 and 0.90, a finding observed in a previous study involving patients with coronary heart disease^(^ 9 ^)^. The item/total correlation analyses, as well as the observation that the removal of items does not significantly improve the Cronbach alpha coefficient, reinforce the homogeneity of the items in each domain. The reliability was also tested through the test-retest, with evidence being obtained of the measure's temporal stability. However, studies involving the application of the SEAMS in other populations, for evaluation of the instrument's measurement properties, were not found in the literature. 

In the present study, evidence of the construct validity of the SEAMS was supported by the analyses of correlation between the SEAMS scores and those of the MMAS-4. However, correlations were not found between the domains of the SEAMS and the measure of general self-efficacy through the GSE. This absence of correlation may be explained by the fact that this scale measures self-efficacy in a generic way, that is, the items of the scale refer to how to deal with success in a specified situation, while the SEAMS evaluate self-efficacy for a specific behavior - medication adherence. However, it is emphasized that negative correlations of moderate to strong magnitude were observed between the SEAMS and the MMAS-4, which suggests convergent construct validity^(^ 9 ^)^.

In relation to the validity of known groups, it was observed that the dimensions and total score of the SEAMS discriminated between CHD patients classified as "adherent" and "non-adherent". Therefore, the sensitivity of the SEAMS, in the detection of differences between the groups, suggests that this instrument may be responsive, that is, capable of measuring changes in self-efficacy for medication adherence, over time. Data were not found in the literature relating to the validity of known groups of the SEAMS.

Self-efficacy is an important construct which can, partly, explain the behavior of medication adherence in CHD patients, as well as being particularly relevant as it is potentially modifiable^(^ 7 ^)^, being able to be the basis for the development of interventions related to behavioral change^(^ 24 ^)^.

The measurement provided by the SEAMS has potential applications for clinical practice and for research. In relation to the clinical implications, this instrument could be used for identifying specific situations, related to the patient's beliefs regarding the perception of her capacity to take the medications, as prescribed by the doctor, which configured challenges for adherence to the medication treatment, in this way making it possible to guide the health professional's actions with a view to strengthening self-efficacy for medication adherence.

As a result, the effectiveness of interventions which strengthen self-efficacy, such as those based in active learning, undertaken through vicarious reinforcement, when the educator shows the patient that other individuals like her are able to adopt the behavior, as well as those of verbal persuasion, in which the professional reinforces that the individual is capable of undertaking such an action, as well as actions directed towards eliminating barriers, must be evaluated through a reliable tool, such as the Brazilian version of the SEAMS. Individuals with high self-efficacy apply greater efforts in coping with barriers, in comparison with those with a low self-efficacy^(^ 25 ^)^.

As a research tool, the measurement of self-efficacy provided by the SEAMS could be a valuable variable of outcome, which could be measured over time in response to a cognitive or educational behavioral intervention, providing evidence regarding the effect of interventions, as well as contributing to a better understanding of the constructs which determine adherence. In this regard, the scale may be used in studies which aim to extend knowledge regarding the mediating and/or moderating variables of this complex behavior. 

As limitations, the absence in the present study of the use of an objective measurement of medication adherence, as well as the use of a generic measurement of evaluation of self-efficacy, are indicated. A review of the literature evidences that none of the measures used for evaluating medication adherence are completely satisfactory, the combined use of objective and subjective measurements of adherence being indicated for this reason^(^ 26 ^)^. Although an objective measurement of medication adherence was not used, it is emphasized that more than one self-reported measure was used, with a view to obtaining a more accurate evaluation of medication adherence. 

As a result, this study provides a tool with evidence of reliability and validity for measuring self-efficacy, for medication adherence, which could be useful in the evaluation of this construct, after nursing interventions directed towards the improvement of self-efficacy for medication adherence.

## Conclusion

This study provides evidence that the Brazilian version of the Self-efficacy for Appropriate Medication Adherence Scale (SEAMS) is an instrument which is easy to understand, and whose measurement properties are reliable and valid. The findings evidence reliability of the total score and of its domains. The construct validity was supported through negative correlations of moderate to strong magnitude between its constructs and the measure of medication adherence (the Brazilian version of the MMAS-4), although evidence was not found for correlations between the Brazilian version of the SEAMS and the general measure of self-efficacy. The validity of known groups was also supported, as the scale is capable of differentiating self-efficacy for adherence among those who were adherent and nonadherent to the medications. However, a high percentage of ceiling effect was observed, suggesting that the Brazilian version of the SEAMS may not be sensitive for detecting improvement in self-efficacy for medication adherence. It is recommended that further studies be undertaken with adaptation of the response scale of the Brazilian version of the SEAMS, and broadening of the sample, with a view to ratifying the findings related to the ceiling effect, as well as to confirm the structure of factors of the Brazilian version of the SEAMS.

## References

[1] American Heart Association (2013). Heart disease and stroke statistics - 2013 update: a report from the
American Heart Association. Circulation.

[2] Zeymer U, Junger C, Zahn R, Bauer T, Bestehorn K, Senges J (2011). Effects of a secondary prevention combination therapy with an aspirin,
an ACE inhibitor and a statin on 1-year mortality of patients with acute
myocardial infarction treated with a beta-blocker. Support for a polypill
approach. Curr Med Res Opin.

[3] Steg PG, James SK, Atar D, Badano LP, Blomstrom- Lundqvist C, Borger MA (2012). ESC Guidelines for the management of acute myocardial infarction in
patients presenting with ST-segment elevation. Eur Heart J.

[4] Haynes RB, Yao X, Degani A, Kripalani S, Garg A, McDonald HP (2005). Interventions for enhancing medication adherence. Cochrane Database Syst Rev.

[5] World Health Organization (2003). Adherence to Long-term Therapies: Evidence for Action.

[6] Leventhal H, Cameron L (1987). Behavioral theories and the problem of compliance. Patient Educ Couns.

[7] Bandura A (1986). Social foundations of thought and action: A social cognitive theory.

[8] Osterberg L, Blaschke T (2005). Adherence to medication. N Engl J Med.

[9] Risser J, Jacobson TA, Kripalani S (2007). Development and Psychometric Evaluation of the Self-Efficacy for
Appropriate Medication Use Scale (SEAMS) in Low-Literacy Patients With Chronic
Disease. J Nurs Meas.

[10] Beaton D, Bombardier C, Guillemin F, Ferraz MB (2007). Recommendations for the Cross-Cultural Adaptation of the DASH and QuickDASH
Outcome Measures.

[11] Mendez RDR, Rodrigues RCM, Cornélio ME, Gallani MCBJ, Godin G (2010). Desenvolvimento de instrumento para medida dos fatores psicossociais
determinantes do comportamento de atividade física em
coronariopatas. Rev Esc Enferm USP.

[12] Morisky DE, Green LW, Levine MA (1986). Concurrent and predictive validity of a self-reported measure of
medication adherence. Med Care.

[13] Ferreira MCS, Gallani MCBJ (2006). Adaptação transcultural do instrumento de Morisky de adesão à
medicação para pacientes com insuficiência cardíaca. Rev Soc Cardiol Estado de São Paulo.

[14] Ventura-Cerdá JM, Mínguez-Gallego C, Fernandez-Villalba EM, Alós-Almiñana M, Andrés-Soler J (2006). Escala simplificada para detectar problemas de adherencia (ESPA) al
tratamiento antirretroviral. Farm Hosp.

[15] Januzzi FF (2009). Qualidade de vida relacionada à função visual e adesão medicamentosa em
idosos com retinopatia diabética.

[16] Schwarzer R, Jerusalem M, Weinman J, Wright, Johnston M (1995). Generalized Self-Efficacy Scale. Measures in health psychology: A user's portfolio - Causal and control
beliefs.

[17] Souza I, Souza MA (2004). Validação da Escala de Autoeficácia Geral Percebida. Rev Univ Rural: Série Ciências Humanas.

[18] Scientific Advisory Committee of the Medical Outcomes Trust (2002). Assessing health status and quality-of-life instruments: attributes
and review criteria. Qual Life Res.

[19] Bennett SJ, Oldridge NB, Eckert GJ, Embree JL, Browning S, Hou N (2002). Discriminant properties of commonly used quality of life measures in
heart failure. Qual Life Res.

[20] Nunnally JC (1978). Psychometric theory.

[21] Fayers PM, Machin D (2000). Quality of life: assessment, analysis and interpretation.

[22] Ajzen I, Fishbein M, Ajzen I, Fishbein M (1980). How to define and measure behavior. Understanding attitudes and predicting social behavior.

[23] Kripalani S, Schmotzer B, Jacobson TA (2012). Improving Medication Adherence through Graphically Enhanced
Interventions in Coronary Heart Disease (IMAGE-CHD): a randomized controlled
trial. J Gen Intern Med.

[24] Baranowski T (1989). Reciprocal determinism at the stages of behavior change: an
integration of community, personal and behavioral perspectives. Int Q Commun Health Educ.

[25] Bartholomew LK, Parcel GS, Kok G, Gottlieb NH (2006). Planning Health Promotion Programs: An Intervention Mapping Approach.

[26] Ye S, Krupka DJ, Davidson KW (2012). Diagnosing medication non-adherence in patient with myocardial
infarction. Front Psychol.

